# Motivations for Pursuing the MRCPsych Qualification Among Psychiatrists: A Cross-Sectional Survey of UK and International Medical Graduates

**DOI:** 10.1192/bjo.2026.11278

**Published:** 2026-06-30

**Authors:** Anju Vivek Shivram, Steve Calvosa

**Affiliations:** 1 South West London and St George's Mental Health NHS Trust, London, United Kingdom; 2 South London and Maudsley NHS Foundation Trust, London, United Kingdom; 3 West London NHS Trust, London, United Kingdom

## Abstract

**Aims:**

The Membership of the Royal College of Psychiatrists (MRCPsych) is a mandatory postgraduate qualification for progression in UK psychiatry training and is widely recognised internationally. International medical graduates make up over 40% of the UK psychiatry workforce. Given recent changes to recruitment processes and policies that prioritise UK medical graduates (UKMGs), it is important to examine motivations for pursuing MRCPsych among both UKMGs and IMGs.

This study aimed to examine motivations for pursuing the MRCPsych qualification among UKMG and IMG psychiatrists, and to explore perceived benefits and challenges among IMGs practising in the UK or Ireland, using a combination of quantitative and qualitative data.

**Methods:**

A cross-sectional survey was conducted among participants attending an online psychiatry teaching programme for the Clinical Assessment of Skills and Competencies examination, delivered via the TresMentis platform by two MRCPsych qualified psychiatrists. Quantitative and qualitative data were collected using structured questions and free text responses. Of 169 responses received, 40 were excluded due to lack of consent, leaving 129 responses for analysis. Variables included country of practice, psychiatric experience, place of primary medical qualification, IMG status, motivations for pursuing MRCPsych, and experiences of working in the UK or Ireland. Qualitative responses were analysed thematically.

**Results:**

Of the 129 respondents, 47.3% were practising in the UK, with others based across South Asia, Africa, the Middle East and Southeast Asia. The majority were IMGs (86.0%), and most were early to mid-career psychiatrists. Common motivations for pursuing MRCPsych included career progression, improvement of psychiatric knowledge and skills, international mobility, and requirements for UK training progression. Qualitative analysis demonstrated clear differences between groups. UKMGs described MRCPsych as a necessary step within a linear UK training pathway. In contrast, IMGs viewed MRCPsych as a flexible and strategic qualification supporting diverse career trajectories, including progression outside formal UK training, recognition of prior experience, and opportunities across different healthcare systems. Among IMGs working in the UK or Ireland, positive experiences included structured training systems, while challenges included adapting to UK healthcare systems, communication barriers and understanding mental health legislation.

**Conclusion:**

MRCPsych is not always pursued for the same reasons by UKMGs and IMGs.While UKMGs primarily undertake the qualification to meet mandatory requirements for progression within UK psychiatry training, IMGs regard MRCPsych as a desirable and versatile credential aligned with a broader range of career options. The desirability of MRCPsych has important implications for workforce planning, training design and recruitment strategies within UK psychiatry.

